# The Development of Facial Bristles in Tawny Frogmouths (*Podargus strigoides*)

**DOI:** 10.1002/dev.70063

**Published:** 2025-07-07

**Authors:** Mariane G. Delaunay, Mark Myers, Carl Larsen, Robyn A. Grant

**Affiliations:** ^1^ Department of Natural Sciences Manchester Metropolitan University Manchester UK; ^2^ Woodland Park Zoo Seattle Washington USA; ^3^ School of Biosciences Liverpool University Liverpool UK

## Abstract

Facial bristles are present in many avian species, although their morphology and function are still not well understood. Previous studies have suggested that rictal bristles are tactile and may play a role in nocturnal foraging, although how they develop and are used is unclear. We study here the facial bristles of the tawny frogmouth (*Podargus strigoides*). We describe the development of rictal bristles, alongside other developmental milestones, such as plumage and eye‐opening. We note four clear stages of plumage and eye‐opening and three stages of rictal bristle emergence. Chicks were born without facial bristles, and rictal bristles emerged after the eyes matured. They were fully developed only after the chick had fledged and engaged in independent feeding. This supports the suggestion that rictal bristles may play a role in independent foraging and feeding.

## 1 Introduction

**FIGURE 1:**
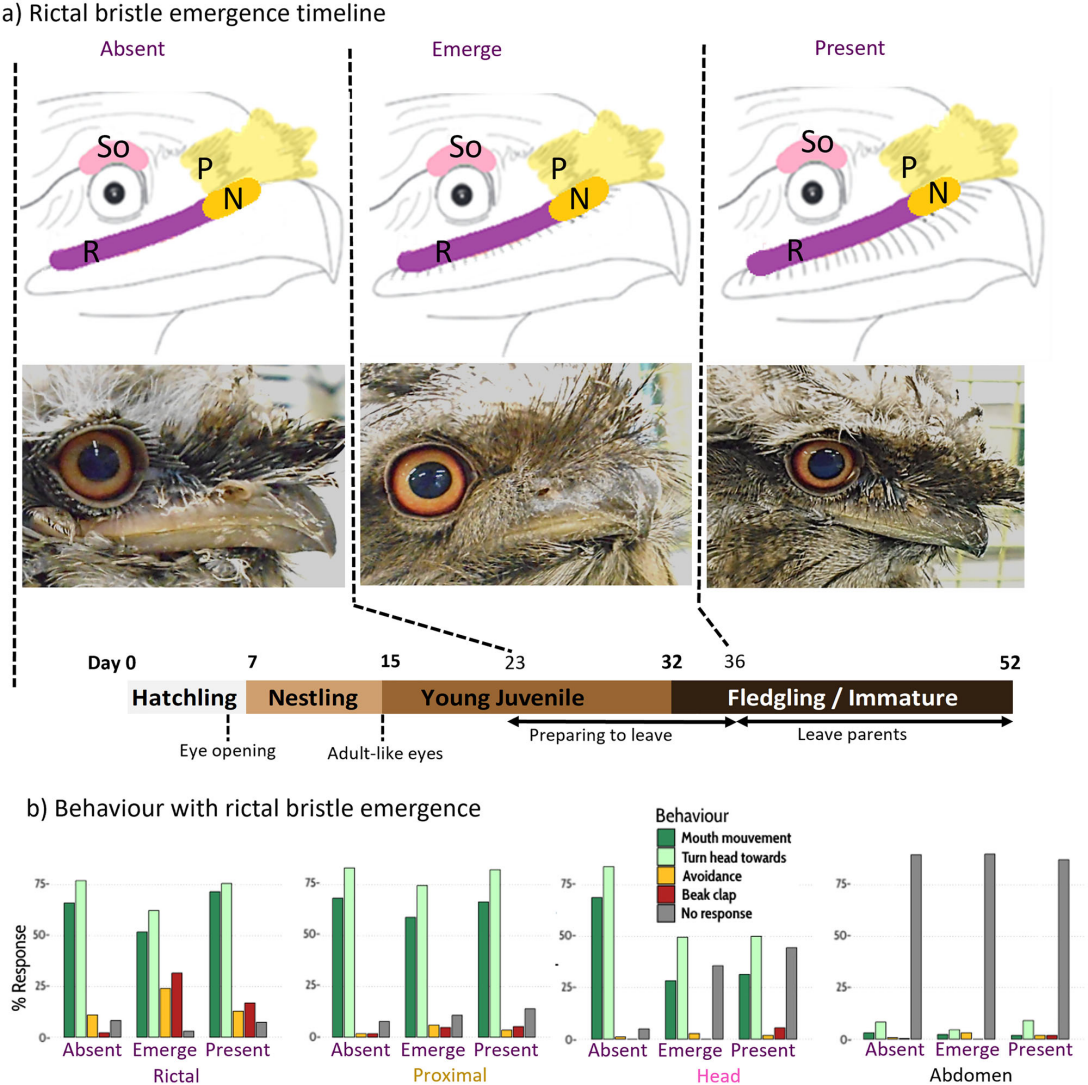
Facial bristles during development of Tawny frogmouth, *P. strigoides*. Panel a shows the timeline of rictal bristle emergence, which takes place over three stages: absent (day 0–22, including the hatchling, nestling, and early juvenile stages), emerging (day 23–35, in the juvenile and early fledgling stages), and present (from day 36, in the fledgling/immature stage). Panel b shows the behaviors associated with touching the bristle areas of the face and the abdomen as a control point, from postnatal day 1–60. Bristle areas include rictal (R), proximal (P) including the narial bristles (N), and the head, including the supraorbital (So) area. Overall, chicks showed less foraging responses (mouth movement and turn head towards) and more no responses when contacted on the abdomen, compared to facial bristle regions. Touches to the rictal bristle area caused more beak claps and avoidance behaviors when the bristles are emerging and present.

Many animals have facial feelers, including bristles on birds and whiskers on mammals. Perhaps the most studied facial feelers are mammalian whiskers (Grant and Goss 2022). Whiskers guide locomotion and foraging, for example, in climbing dormice (Arkley et al. 2017) and cricket‐eating Etruscan shews (Anjum et al. 206). Whiskers tend to be more moveable and sensitive in mammals that forage in dark, complex habitats, such as pinnipeds and scansorial rodents (Grant and Goss 2022; Muchlinski et al. 2020; Milne et al. 2022; Grant et al. 2018). Compared to mammalian whiskers, avian bristles are relatively understudied. They include, but are not limited to, the rictal, supraorbital, and narial bristles (Figure 1a). Although they may superficially resemble mammalian whiskers, the function of avian bristles is not fully known. Recent work by Delaunay et al. (2020) suggested that Caprimulgiform species that forage in closed and complex habitats at night are more likely to have mechanoreceptors around their rictal bristles. Furthermore, a large study of 1022 bird species found that rictal bristle presence and length were associated with species ecology, especially nocturnality (Delaunay et al. 2022). Specifically, species foraging in low‐light conditions were more likely to possess longer rictal bristles. However, experimental studies do not always provide evidence of use during foraging. For instance, it was observed that insectivorous Neotropical tyrant‐flycatchers captured prey items between the tip of the mandibles without the prey making any contact with the bristles (Conover and Miller 1980; Lederer 1972).

As well as being involved in guiding locomotion and foraging, mammalian whiskers also play important roles during development, including finding the mother's nipple for feeding (Sullivan et al. 2003), maintaining contact with conspecifics for huddling and thermoregulation (Grant, Sperber, et al. 2012), as well as for orienting toward contact (Grant, Sperber, et al. 2012). Indeed, rats, for instance, are born with whiskers, which are likely to be functional at birth, and even moveable from day 2 (Grant, Mitchinson, et al. 2012). Our study builds on the classic rat pup whisker stimulation work of Sullivan et al. (2003), who found that, from as early as day 3, stimulating the whiskers with a wooden rod caused heightened activity in young rat pups. Specifically, they responded to whisker stimulation with head raising, mouthing, and turning towards or away from the stimulus, behaviors that are particularly associated with nipple search and attachment. Nothing is known about the role of avian facial bristles during development, nor even if they are present from hatching.

Here, we focus on tawny frogmouths (*Podargus strigoides*; order: Caprimulgiformes; family: Podargidae) and describe their rictal bristle development. We have already described the rictal bristle follicle anatomy of adult tawny frogmouths and found Herbst corpuscles (mechanoreceptors) (Figure S1d) around the follicles of their rictal bristles, numbering around four per follicle (Delaunay et al. 2020). Previously unpublished data from our lab (shown in Figure S1) show Herbst corpuscles are also present around the supraorbital and narial bristle follicles, numbering around three and four, respectively. This suggests that all three facial bristles are likely to be tactile, in the adult bird at least. We describe here the developmental schedule of tawny frogmouths, particularly documenting the development of rictal bristle emergence. The chicks’ behavioral responses to facial bristle touch are also analyzed from birth to day 52 (fledging/immature stage). If bristles do function similarly to rat whiskers, then they may well be present and functional in hatchlings.

## 2 Materials and Methods

### 2.1 Animals

The Woodland Park Zoo in Seattle, USA, keep breeding pairs of *P. strigoides*, which have chicks every year. Selected chicks were hand‐reared, while others remained with parents for rearing. Data were collected over a period of 3 years (2017–2019). In total, nine *P. strigoides* chicks were included in this study, with data from four to six individuals each day. All work was carried out in accordance with the local ethics committee at Manchester Metropolitan University.

### 2.2 Describing the Developmental Schedule

*Podargus strigoides* chicks were taken from their enclosures individually, rested on a surface for around 1 min and filmed using a GoPro Hero 5 camera (60 fps). All videos were studied to document the developmental schedule of the facial bristles in relation to feather development and eye‐opening. Since the rictal bristles are more prominent and numerous (numbering around 10, compared to around five in other bristle types), these became the focus of the facial bristle emergence description. Four clear development periods, based on the observations of four body feather phenotypes, were defined here as hatchling, nestling, young juvenile, and fledgling/immature (Figure 1a). Hatchlings were covered with white down feathers. The nestling stage corresponded to the emergence of gray/brown feathers superseding the white down feathers. In the young juvenile stage, the white down feathers disappeared completely superseded by the juvenile feathers. In the fledgling/immature stage, all juvenile feathers were molted, and only adult feathers covered the body.

### 2.3 Characterization of Behavioral Responses to Touch

Each individual was touched during filming using a cotton bud on four points including: (i) the top of the head (including the supraorbital region), (ii) rictal region, (iii) proximal region (the skin region directly above the beak and between the eyes, central to the face, which is likely to touch the narial region) (Figure 1a), and (iv) abdomen area (as a control point). Each touch point was repeated three times for each chick per day, with a pause of ∼2 s between subsequent touches to give the chick time to settle. The total time to complete the task was less than a minute per day. All videos were reviewed, and a single observer scored them according to a standardized and validated ethogram (Table 1; see Supporting Information 2, Table S1 for validation). The ethogram (Table 1) was developed specifically for this study and was based on three categories of behaviors, which were foraging, avoidance, and annoyance. For each chick per day, the behavioral response to the cotton bud touch was counted as present or absent at each touch point position. Therefore, for each chick, the total number of occurrences of a behavioral response was recorded at each touch point position for that day, along with the number of touches, for example, the total number of times the cotton bud touched a given touch point position per chick and per video.

**TABLE 1:** Ethogram with descriptions of observed behavioral responses in response to touch with a cotton bud, throughout the development of *Podargus strigoides*.

Categories	Behavior	Description (as observed in videos)
Foraging	Mouth movement	The bird opens and closes its mouth
	Bite	The bird catches the cotton bud in mouth
	Swallow	The bird tries to ingest the cotton bud once caught, with a throat movement
	Lift body up	The bird lifts its body and head upward without moving its feet, i.e., no displacement
	Turn head towards	The bird turns its gaze and head towards the cotton bud
Avoidance	Shake head	The bird moves its head from side to side
	Retract head	The bird lowers its head down into its shoulders or slightly backward, away
	Avoid	The bird leans its whole body backward or sideways, away
	Wing movement	The bird moves its wing up and down in a stretch or a rapid movement, i.e., twitch
	Step away	The bird moves or shuffles away, making at least one step
Annoyance	Beak clap	The bird makes a clicking sound with its beak by opening and closing it, sometimes its tongue is out
No response	No response	The bird shows no response

### 2.4 Statistical Analysis

The effect of age (day 1–52), touch point position (top of the head, abdomen, rictal region, and proximal region), rictal bristle emergence (absence, emergence, present), and eye‐opening stages (closed, open 1, open 2, open 3) on the behavioral responses were investigated (Supporting Information 3, Figure S2). Age, touch point position, rictal bristle emergence, and eye‐opening stages were included as separate fixed effects in a linear mixed effects model, with the chick ID as a random effect. While the focus of this study is on rictal bristle emergence, eye opening is another key developmental stage that may affect the same behaviors, and results of which can be seen in Supporting Information 4, Table S4. All models were constructed using the *lmer* function (lme4 package; Bates et al. 2015) in the R Studio software (R Core Team 2018) (see Supporting Information 4, Tables S2–S4). *lmer* was selected as it accounts for inter‐individual variability in behavioral responses by including random effects. Moreover, the data were not normally distributed (checked using Shapiro–Wilk test; Shapiro and Wilk 1965) and had missing data points, but *lmer* is flexible and can accommodate both nonparametric data and missing data points, so that chicks with data for at least one touch point could still be included in the analysis. Of the 12 behaviors (Table 1), only behaviors occurring more than 30 times were used in the models. The behaviors shake head, retract head, avoid, wing movement, and step away (Table 1) were merged as avoidance behaviors for the analysis and corrected with the total number of times the region was touched (i.e., maximum of times the behavior could have occurred). In total, seven behaviors were included in the analysis, including mouth movement, turning head toward the stimulus, avoidance (including shakes head, retracts head, avoids, wing movement, and steps away), beak clapping, and no response (Table 1; Figure 1b). To visualize the data, the percentage of occurrences of each behavior (the sum of occurrences per behavior was divided by the sum of number of contacts) was calculated and then plotted against different fixed effects (touch point positions, development of feathers, rictal bristles emergence, and eye‐opening stages), using *ggbarplot* (ggpubr package; Wickham 2016).

## 3 Results

### 3.1 Developmental Schedule

We observed three clear stages of rictal bristle emergence and four different stages of eye‐opening (Supporting Information 3, Figure S2). The hatchling stage occurs from birth to day 7, during which time rictal bristles are absent. The hatchling opens its eyes around day 6–7 (Figure 1a). At this stage, their eyes are dark and small with no colored iris visible (Supplement 3, Figure S2 “open 1”). The nestling stage is from day 7–8 to day 14 (Figure 1a). During this stage, the eyes grow, and a colored iris appears around day 11 (Supplement 3, Figure S2 “open 2”). Next, the chick enters the young juvenile stage, attaining the final stage of eye‐opening on day 15, which is adult‐sized eyes with clear colored irises and the appearance of eyelashes around the eyes (Supplement 3, Figure S2 “open 3”). The young juvenile chick also displays a body covered with gray and brown body feathers, with the total disappearance of white down feathers from day 15 to 31. The rictal bristles start to emerge from day 23. A small outgrowth of skin (the papilla) emerges in the rictal region from which the rictal bristle starts to grow (Figure 1a). The fledgling rictal bristles finish their development, resembling fully formed adult bristles, around day 35 (Figure 1a).

### 3.2 Behavioral Responses to Touch

*Podargus strigoides* chicks exhibit more foraging behaviors (including mouth movement, bite, swallow, turn head toward) when stimulated by touch on the rictal (84.47%), proximal (86.69%), and head (81%) regions, whereas they have less foraging responses (10.13%) and more unresponsiveness (89.45%) when touched on the abdomen. Age had a significant effect on beak clapping behaviors (*p* < 0.001). Indeed, as rictal bristles develop (emerge and present) when the chicks are young juveniles with no white down feathers, both avoidance and beak clap behaviors increase (*p* = 0.045 and *p* < 0.001, respectively; Table S2, Figure 1b). Touches to the rictal bristles cause more beak claps as the bristles develop, compared to touches on any other regions (*p* < 0.001; Table S2, Figure 1b), and more avoidance behaviors than touches to the proximal region (*p* = 0.013; Table S2, Figure 1b). As the bristles emerge, touches to the rictal bristle area cause more mouth movements and head turns toward the cotton bud, compared to touches on the head (*p* < 0.001; Table S2, Figure 1b).

## 4 Discussion

If bristles function similarly to rat whiskers, they should be present and useable in birds from hatching. However, this is not the case, and *P. strigoides* chicks are born without any facial bristles. Rictal bristles emerge after the eyes are mature and are fully developed after the chick has fledged. This lends support to the hypothesis that rictal bristles may play a role in independent foraging in fledged *P. strigoides* chicks.

### 4.1 Developmental Schedule of Podargus Strigoides

Hatchling chicks (day 0–7) have white down feathers, absent rictal bristles, and closed eyes. Nestling chicks (day 7–15) have some gray/brown body feathers replacing the white down feathers. The chicks’ eyes mature during the nestling period and are adult‐like from day 15, around 8 days before the rictal bristles emerge. Therefore, *P. strigoides* might rely more on vision than touch as young chicks, and rictal bristles are unlikely to play a major tactile role in developing chicks. Young juvenile chicks (day 15–32) are covered with gray and brown body feathers, without any white down feathers. Their eyes are adult‐like and in this stage rictal bristles start to emerge. Fledgling/immature chicks (day 32–52) have complete adult plumage, their rictal bristles become adult‐like, and they also start to feed independently at this time.

### 4.2 Behavioral Responses to Touch

Behavioral responses to rictal region touches manifest as foraging behaviors, including mouth movements and turning head toward the cotton bud, much like the behaviors described by Sullivan et al. (2003) in rat pups. These foraging behaviors, along with avoidance and annoyance (e.g., beak clap) behaviors, are triggered more when chicks are touched on the rictal region, especially as the bristles develop. This suggests that rictal bristles are touch‐sensitive, in agreement with Delaunay et al. (2022), and, unlike rat whiskers that are functional from birth, become more functional as the chicks develop. However, as the chick gets older, it also responds less to the stimulus, likely becoming accustomed to the touch, and learning that there is no food reward associated with it. Touches to the head (around the supraorbital) and the proximal area (near the narial bristles) also cause many foraging behaviors, especially compared to abdomen touches. Therefore, all facial bristles are likely to be tactually sensitive, which is also supported by the presence of mechanoreceptors around their follicles in adult animals (Supplement 1, Figure S1). Foraging and head turning behaviors were prevalent following bristle region touches, even when the rictal bristles were entirely absent (Figure 1b). These skin areas will still be sensitive to touch, and these behaviors might be important to guide parent–offspring feeding interactions, which occur in the nest at night in young chicks (Körtner and Geiser 1999). However, that the facial bristles emerge as the chick starts to forage independently likely indicates that the bristles do not play a role in parent–chick feeding but are probably more associated with independent foraging or locomotion. In the future, investigating the role facial bristles play in fledgling *P. strigoides*, especially in foraging, avoidance, or protection of the eyes and nostrils from debris and soiling, would be beneficial.

### 4.3 Conclusions

*Podargus strigoides* chicks are born without any facial bristles. Full rictal bristle emergence coincides with when the chicks started to feed independently as fledglings, suggesting that these bristles may play a role in foraging, including guiding orienting to food and avoiding collisions. We suggest that facial bristles play a role in feeding in older chicks and adults. This is unlike whiskers which are present and functional in rats from birth.

## Conflicts of Interest

The authors declare no conflicts of interest.

## Supporting information




Supplement 1: Follicle anatomy of facial bristles
Figure S1: Example slices through facial bristle follicles in adult *Podargus strigoides* including a) supraorbital (eyebrow), b) narial and c) rictal bristles. Slices are 10 µm and stained with Masson's trichrome. d) follicle; *: muscle fibres; Arrows: Herbst corpuscles. Scale bars are 200 µm in panels a‐c and 20 µm in panel d.
Supplement 2: Ethogram Reliability Analysis
Table S1. Intra‐ and inter‐observer reliability analysis scoring four videos of *P. strigoides* chicks during their development.
Supplement 3: Eye opening and bristle emergence timeline
Figure S2. Timelines figure representing the different stages of development of the *Podargus strigoides* chicks. The rictal bristle emergence and eye‐opening are illustrated on the timeline. Rictal bristle emergence can be scored as absent, emerging and fully developed. Eye opening can be described as closed, and three stages of eye‐opening
Supplement 4: Statistical Analysis
Table S2. Summary of the effects of rictal bristle (RB) emergence and touch point positions on *Podargus strigoides* chicks’ behavioural responses, with absence of rictal bristles and touch on the rictal region as reference. Significant p‐values are indicated with an asterisk.
Table S3 Summary of the effects of touch point positions (model A) and age (model B) on the *Podargus strigoides* chicks’ behavioural responses, with day 0 and touch on the rictal region as reference. Significant p‐values are indicated with an asterisk.
Table S4. Summary of the effects of eye‐opening and touch point positions on *Podargus strigoides* chicks’ behavioural responses, with closed eyes and touch on the rictal region as reference. Significant p‐values are indicated with an asterisk.

## Data Availability

All summary data can be seen in figure 1 and all statistical data can be found in the Supplementary material. Videos and raw data can be provided by the authors.
