# Supplementary material for: The Development of Facial Bristles in Tawny Frogmouths (Podargus strigoides)
Source: Dev Psychobiol. 2025 Jul 7;67(4):e70063. doi: 10.1002/dev.70063 (PMC12230641; doi:10.1002/dev.70063)
Supplement: Supplementary file 1 — Supplement 1: Follicle anatomy of facial bristles Figure S1: Example slices through facial bristle follicles in adult Podargus strigoides including a) supraorbital (eyebrow), b) narial and c) rictal bristles. Slices are 10 µm and stained with Masson's trichrome. d) follicle; *: muscle fibres; Arrows: Herbst corpuscles. Scale bars are 200 µm in panels a‐c and 20 µm in panel d. Supplement 2: Ethogram Reliability Analysis Table S1. Intra‐ and inter‐observer reliability analysis scoring four videos of P. strigoides chicks during their development. Supplement 3: Eye opening and bristle emergence timeline Figure S2. Timelines figure representing the different stages of development of the Podargus strigoides chicks. The rictal bristle emergence and eye‐opening are illustrated on the timeline. Rictal bristle emergence can be scored as absent, emerging and fully developed. Eye opening can be described as closed, and three stages of eye‐opening Supplement 4: Statistical Analysis Table S2. Summary of the effects of rictal bristle (RB) emergence and touch point positions on Podargus strigoides chicks’ behavioural responses, with absence of rictal bristles and touch on the rictal region as reference. Significant p‐values are indicated with an asterisk. Table S3 Summary of the effects of touch point positions (model A) and age (model B) on the Podargus strigoides chicks’ behavioural responses, with day 0 and touch on the rictal region as reference. Significant p‐values are indicated with an asterisk. Table S4. Summary of the effects of eye‐opening and touch point positions on Podargus strigoides chicks’ behavioural responses, with closed eyes and touch on the rictal region as reference. Significant p‐values are indicated with an asterisk. [file DEV-67-e70063-s001.pdf]

Supplementary Material

Supplement 1: Follicle anatomy of facial bristles

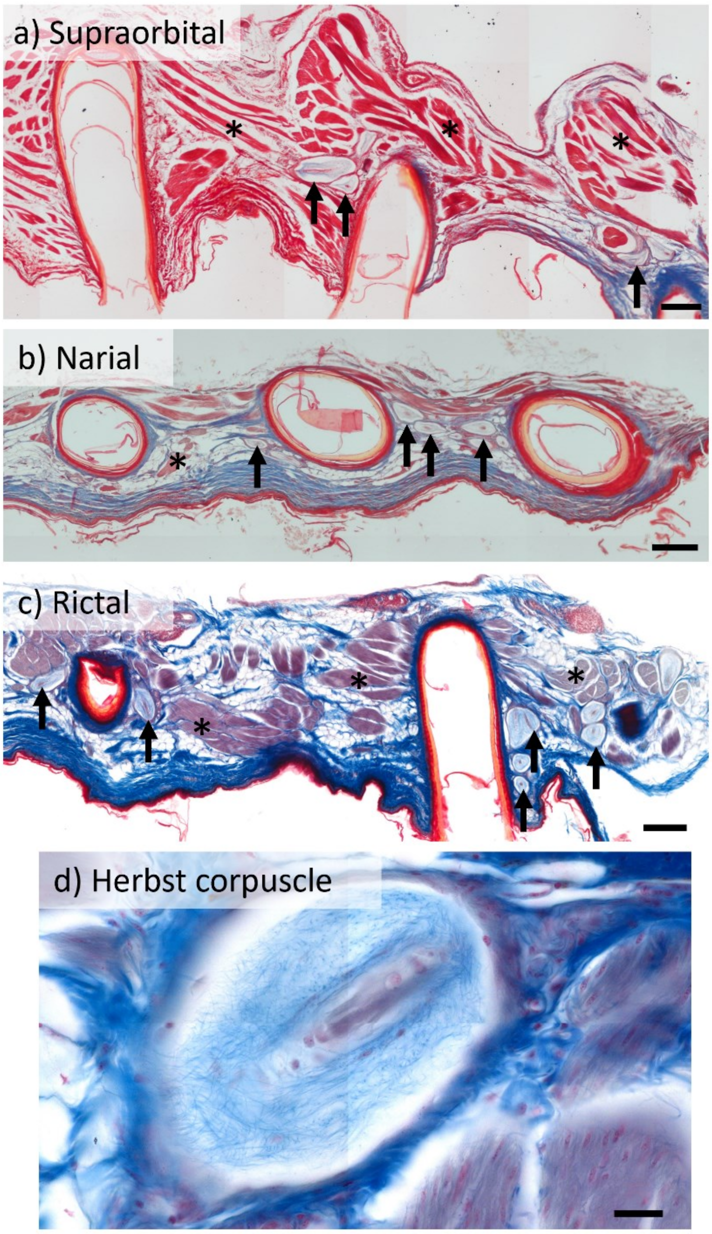

Figure S1: Example slices through facial bristle follicles in adult *Podargus strigoides* including a) supraorbital (eyebrow), b) narial and c) rictal bristles. Slices are 10 µm and stained with Masson's trichrome. d) follicle; *: muscle fibres; Arrows: Herbst corpuscles. Scale bars are 200 µm in panels a-c and 20 µm in panel d.

Supplement 2: Ethogram Reliability Analysis

To assess the reliability of the ethogram, four videos for each species at the four different stages of chick development were chosen at random from the dataset. Six behavioural responses were tested, including mouth movement, look towards, turn beak towards, avoidance, beak clap and no response, for each of the touch point position. The videos were scored once again by the same single observer to obtain the intra-reliability score, and again by one other observer to obtain the inter-observer reliability. Scores were then compared using Cronbach's α (Table S1). As shown in the table, all reliability scores were greater than 0.8 indicating that all six measures had a high internal consistency as per Cronbach (1951). Subsequently, the ethogram is robust enough to be scored by a single observer.

Table S1. Intra- and inter-observer reliability analysis scoring four videos of *P. strigoides* chicks during their development.

Behaviour	Cronbach's α scores	
	Intra-observer	Inter-observer
Mouth movement	0.95	0.93
Turn head towards	0.99	0.82
Avoidance	0.97	0.98
Beak clap	1	1
No response	0.89	0.96

Supplement 3: Eye opening and bristle emergence timeline

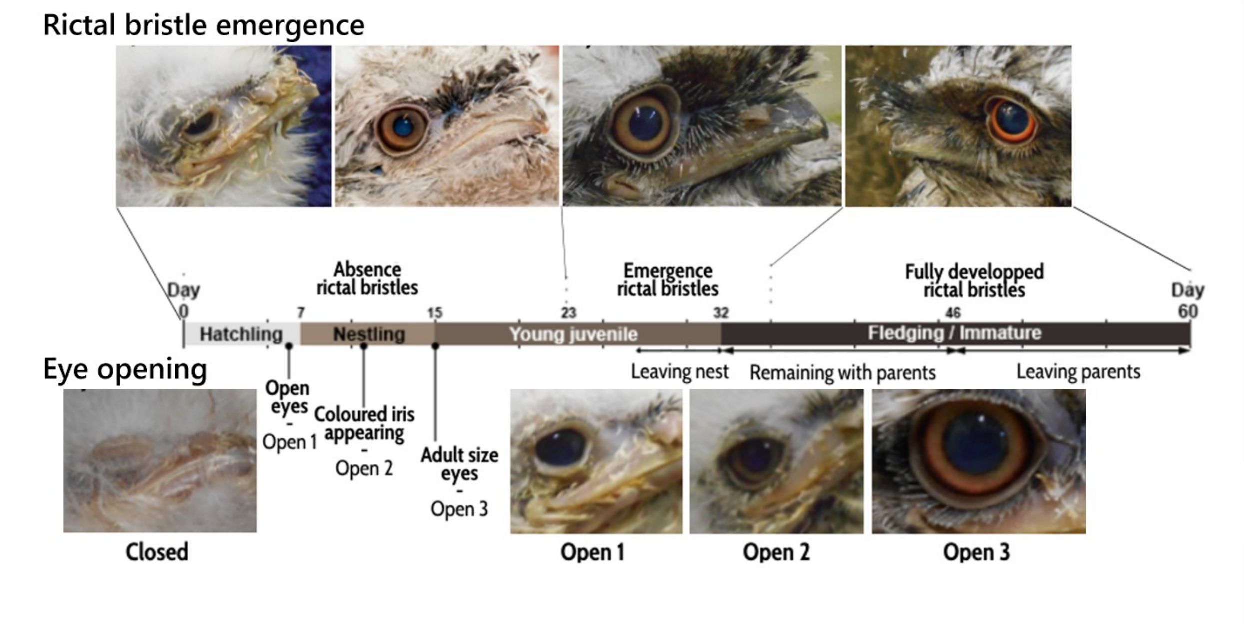

Figure S2. Timelines figure representing the different stages of development of the *Podargus strigoides* chicks. The rictal bristle emergence and eye-opening are illustrated on the timeline. Rictal bristle emergence can be scored as absent, emerging and fully developed. Eye opening can be described as closed, and three stages of eye-opening.

Supplement 4: Statistical Analysis

Table S2. Summary of the effects of rictal bristle (RB) emergence and touch point positions on *Podargus strigoides* chicks' behavioural responses, with absence of rictal bristles and touch on the rictal region as reference. Significant p-values are indicated with an asterisk.

Behaviour / Tests	Mouth movement		Turn head towards		Avoidance		Beak clap		No response	
	Estimates	*p*-value	Estimates	*p*-value	Estimates	*p*-value	Estimates	*p*-value	Estimates	*p*-value
**Intercept**	1.58	<0.001*	1.95	<0.001*	0.43	<0.001*	0.13	<0.001*	0.20	0.015*
**RB dev**	0.11	0.202	0.01	0.936	0.11	0.045*	0.25	<0.001*	-0.00	0.966
**Proximal**	-0.80	<0.001*	-0.91	<0.001*	-0.16	0.051	-0.10	0.034*	-0.09	0.328
**Head**	-0.30	0.013*	-0.27	0.041*	0.00	0.984	-0.14	0.006*	-0.03	0.766
**Abdomen**	-1.57	<0.001*	-1.76	<0.001*	-0.40	<0.001*	-0.13	0.011*	1.78	<0.001*
**RB dev * Proximal**	-0.08	0.467	-0.00	0.982	-0.19	0.013*	-0.23	<0.001*	0.06	0.531
**RB dev * Head**	-0.60	<0.001*	-0.56	<0.001*	-0.12	0.133	-0.23	<0.001*	0.24	0.008*
**RB dev * Abdomen**	-0.05	0.635	0.01	0.955	-0.11	0.163	-0.25	<0.001*	-0.39	<0.001*
**Number of observations**	1000		1000		1000		1000		1000	

Table S3 Summary of the effects of touch point positions (model A) and age (model B) on the *Podargus strigoides* chicks' behavioural responses, with day 0 and touch on the rictal region as reference.. Significant p-values are indicated with an asterisk.

Behaviour / Tests	Mouth movement		Turn head towards		Avoidance		Beak clap		No response	
	Estimates	*p*-value	Estimates	*p*-value	Estimates	*p*-value	Estimates	*p*-value	Estimates	*p*-value
**Intercept**	1.61	<0.001*	2.13	<0.001*	0.40	<0.001*	-0.03	0.609	0.26	0.015*
**Age**	0.00	0.818	-0.01	0.068	0.01	0.105	0.02	<0.001*	-0.00	0.453
**Proximal**	-0.89	<0.001*	-1.12	<0.001*	-0.14	0.242	0.04	0.574	-0.20	0.143
**Head**	-0.00	0.999	-0.00	0.999	0.04	0.755	0.01	0.834	-0.26	0.061
**Abdomen**	-1.62	<0.001*	-1.93	<0.001*	-0.38	0.001*	0.04	0.616	1.80	<0.001*
**Age * Proximal**	0.00	0.779	0.01	0.175	-0.01	0.123	-0.01	<0.001*	0.01	0.208
**Age * Head**	-0.03	<0.001*	-0.03	<0.001*	-0.00	0.686	-0.01	<0.001*	0.02	0.001*
**Age * Abdomen**	0.00	0.960	0.01	0.262	-0.00	0.334	-0.02	<0.001*	-0.01	0.017*
**Number of observations**	1000		1000		1000		1000		1000	

Table S4. Summary of the effects of eye-opening and touch point positions on *Podargus strigoides* chicks' behavioural responses, with closed eyes and touch on the rictal region as reference. Significant p-values are indicated with an asterisk.

Behaviour / Tests	Mouth movement		Turn head towards		Avoidance		Beak clap		No response	
	Estimates	*p*-value	Estimates	*p*-value	Estimates	*p*-value	Estimates	*p*-value	Estimates	*p*-value
**Intercept**	1.65	<0.001*	2.41	<0.001*	0.48	<0.001*	0.00	1.000	0.61	<0.001*
**Eye Dev – open 1**	0.49	0.056	-0.15	0.571	-0.10	0.567	0.00	0.999	-0.62	0.001*
**Eye Dev – open 2**	0.26	0.301	-0.36	0.177	-0.23	0.172	0.00	0.998	-0.61	0.002*
**Eye Dev – open 3**	-0.20	0.307	-0.78	<0.001*	0.25	0.052	0.57	<0.001*	-0.45	0.002*
**Eye Dev – open 4**	0.01	0.983	-0.63	0.028*	-0.30	0.096	0.16	0.135	-0.36	0.078
**Proximal**	-1.02	<0.001*	-1.43	<0.001*	-0.43	0.005*	0.02	0.795	0.50	0.004*
**Head**	-0.36	0.115	-0.45	0.061	-0.31	0.042*	0.00	1.000	-0.57	0.001*
**Abdomen**	-1.60	<0.001*	-2.29	<0.001*	-0.45	0.003*	0.02	0.795	0.86	<0.001*
**Open 1 * Proximal**	-0.22	0.539	0.22	0.558	0.26	0.283	-0.02	0.868	0.64	0.018*
**Open 2 * Proximal**	-0.04	0.903	0.40	0.291	0.63	0.008*	0.01	0.946	0.50	0.061
**Open 3 * Proximal**	0.48	0.070	0.92	0.001*	0.01	0.958	-0.53	<0.001*	0.56	0.005*
**Open 4 * Proximal**	-0.34	0.365	-0.09	0.817	0.31	0.216	-0.18	0.220	0.34	0.227
**Open 1 * Head**	-0.19	0.583	0.25	0.516	0.31	0.194	-0.00	1.000	0.67	0.012*
**Open 2 * Head**	0.19	0.587	0.29	0.445	0.54	0.022*	-0.00	1.000	0.57	0.032*
**Open 3 * Head**	-0.46	0.081	-0.27	0.342	0.22	0.210	-0.55	<0.001*	1.05	<0.001*
**Open 4 * Head**	-1.16	0.002*	-1.19	0.003*	0.23	0.357	-0.16	0.285	0.37	0.187
**Open 1 * Abdomen**	-0.47	0.182	0.39	0.303	0.07	0.759	-0.02	0.868	0.83	0.002*
**Open 2 * Abdomen**	-0.40	0.249	0.35	0.346	0.25	0.285	-0.02	0.866	1.71	<0.001*
**Open 3 * Abdomen**	0.18	0.502	0.77	0.006*	-0.19	0.274	-0.59	<0.001*	0.90	<0.001*
**Open 4 * Abdomen**	0.04	0.924	0.57	0.153	0.33	0.183	-0.18	0.220	-0.86	0.002*
**Number of observations**	1000		1000		1000		1000		1000	
